# Cross-Linked Phosphorylated Cellulose as a Potential Sorbent for Lithium Extraction from Water: Dynamic Column Studies and Modeling

**DOI:** 10.1021/acsomega.2c04712

**Published:** 2022-10-21

**Authors:** Yaşar Kemal Recepoğlu, Aslı Yüksel

**Affiliations:** † Department of Chemical Engineering, Izmir Institute of Technology, 35430Urla, Izmir, Turkey; ‡ Geothermal Energy Research and Application Center, Izmir Institute of Technology, 35430Urla, Izmir, Turkey

## Abstract

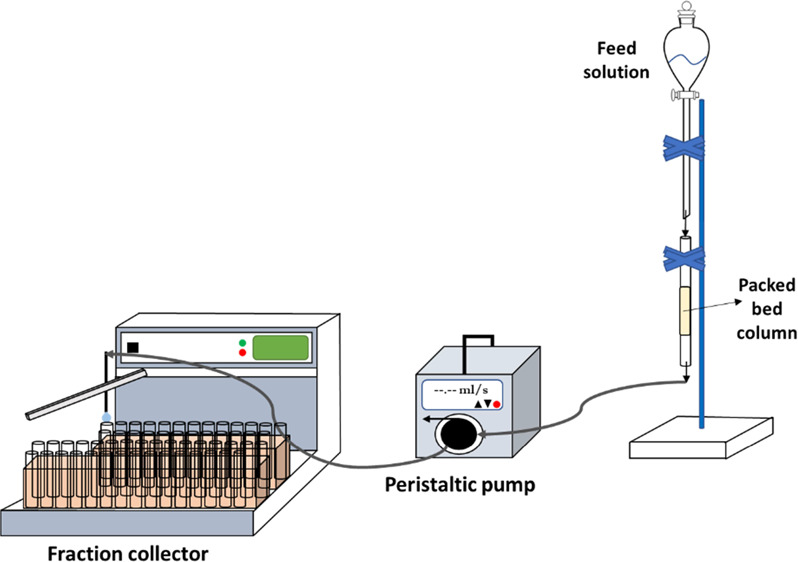

Phosphorylated functional cellulose was cross-linked with epichlorohydrin at different ratios because it is a very hydrophilic substance that instantly swells to form a hydrogel when it comes into contact with water. It was aimed to utilize a continuously packed bed column to recover lithium from water under varying operating conditions such as flow rate and bed height. The characterization results confirmed cross-linking based on morphology, structure, surface area, and thermal stability differences. Lithium recovery was more efficient with a low flow rate, but the dynamic sorption process was independent of bed height. The total capacities at the three flow rates with 1.5 cm bed height were 33.56, 30.15, and 25.54 mg g^–1^, and the total saturation times at the three different bed heights with 0.5 mL min^–1^ flow rate were 659, 1001, and 1007 min, respectively. Only 15.75 mL of 5% H_2_SO_4_ solution was required to desorb approximately 100% of Li from the saturated sorbent.

## 1 Introduction

As new renewable energy technologies, such as electric vehicles and energy storage systems, emerge, the need for lithium salts is also expected to rise dramatically.^1,2^ The use of lithium as an electrode material in rechargeable batteries has been a significant market opportunity since the 1990s.^3^ Lithium is obtained primarily from rocks as a mineral and liquid resources; however, the quality of lithium ores has deteriorated due to over-mining. Thus, lithium recovery from aqueous sources, such as seawater, geothermal water, and salt-lake brines, has become an essential option for lithium salt production.^4,5^ The solar evaporation process is the most widely used industrial method for recovering Li from brines.^6^ Several potential strategies, including adsorption,^7−9^ solvent extraction,^10−13^ and membrane-related technologies,^14−16^ for Li recovery from brines, have been reported to replace solar evaporation because it needs a long time to concentrate considerable amounts of Li. Adsorption is the most promising strategy for recovering Li from aqueous resources because it is more climate-friendly and more effective in an industrial application.^17^ Although there are many studies reported in the literature regarding metal ion extraction based on different functionalized composite materials,^18−26^ aluminum hydroxides (AlOH),^27,28^ aluminum oxides (AlO_*x*_),^29^ manganese oxides (MnO_*x*_),^30−34^ and titanium oxides (TiO_*x*_)^4,35−37^ have been known to be the most selective lithium adsorbents until now. They are classified as inorganic crystalline solids either being studied for direct lithium extraction from brines or employed as cathode materials in lithium-ion batteries. Furthermore, researchers have been looking at using selective cation-exchange resins as organic sorbents in the form of a strong acid to recover lithium from lithium-containing fluids. Lewatit K2629, TP 207, TP 208,^38^ and Lewatit TP 260^39^ are some organic sorbents exhibiting low selectivity of lithium because they only become efficient when impregnated with inorganic ones that have just been aforementioned. Ion-imprinted polymers are developed as organic sorbents to separate lithium selectively from an aqueous medium.^40^ To our best knowledge, lithium recovery using low-cost and sustainable sources containing a considerable portion of cellulose is a new field with scant literature. Since the development of the next generation of materials has been critical in terms of sustainability and green chemistry over the last decade, bio-based polymer matrices potentially allow for lower environmental impacts through the use of renewable biomass and biodegradable or reusable materials are of great interest.^41,42^ Moreover, the synthesis and development of innovative functional low-cost adsorbents with large capacity, high selectivity, and high adsorption rate continue to pique attention for both hazardous substance removal and mineral recovery from water.^43^ Cellulose is the most common natural polymer, accounting for 35–50% of all plant materials on the planet.^44^ It is a linear syndiotactic photopolymer made up of -anhydroglucopyranose units (AGU) connected by β-(1 → 4)-glucosidic linkages. At both ends of the cellulose chain, the hydroxyl groups function differently. Intermolecular and intramolecular interactions such as hydrogen bonding and degradation processes mainly include bridging and ring oxygen atoms.^45^ The presence of hydroxyl groups on cellulose enables several modification processes to create new sorbents with varied functional groups.^46^ Phosphorylation is one approach to modifying cellulose. The chelating properties of phosphate groups are well recognized. As a result, phosphorylated polysaccharides have been exploited as metal-chelating polymers and cation exchange materials for pollution remediation.^42^ Recently, microcrystalline cellulose along with hazelnut shell waste and olive pruning waste as natural cellulose resources has been phosphorylated to synthesize lithium-capable low-cost and sustainable sorbents and tested via batch mode sorption studies in our research group.^1,47,48^

A batch mode, known as static adsorption, or a continuous flow mode, known as dynamic adsorption in packed bed columns, can be used to design and operate an adsorption system. The material itself merely acts as an adsorbent in batch tests, and metal ions are adsorbed mainly by the surface area of its outer layers, reducing the adsorbent’s efficacy.^49^ However, the material performs both filtration and adsorption in dynamic adsorption. Low adsorption rates due to the bulk of binding sites situated inside the pores of the adsorbents and high-pressure drops caused by tightly packed small particles in a column are common difficulties for the traditionally packed bed column. However, column adsorption is still more suitable in large-scale operations in the industry than batch adsorption because it is more straightforward, requiring no additional operations such as filtration or centrifugation.^34,43,50^ Therefore, in this study, phosphorylated functional cellulose (FC) was used for the first time in a dynamic packed bed column to recover lithium from water under varying operating conditions such as bed height and flow rate. However, before utilization, it was first cross-linked with epichlorohydrin (ECH) at different ratios (0.02, 0.04, and 0.08 mL ECH/g FC) because it is a very hydrophilic substance that instantly swells to form a hydrogel when it comes into contact with water. The synthesized material’s textural, structural, and thermal properties were investigated. The effects of the flow rate and bed height on breakthrough curves and the saturated column’s reusability by packed bed dynamic studies were studied extensively. After all, the breakthrough curves were fitted to commonly used models for a fixed-bed column such as Thomas, Yoon–Nelson, and modified dose–response (MDR) models to estimate relative factors like the breakthrough time and the adsorption capacity.

## 2 Experimental Section

### 2.1 Materials

Chemicals used in this study included sodium chloride (NaCl, CP, 99.5%), lithium chloride (LiCl, AR, 99%), potassium chloride (KCl, AR, 99%), ECH (C_3_H_5_ClO), sulfuric acid (H_2_SO_4_, 95–97%), and potassium hydroxide (KOH) and were purchased from Merck.

### 2.2 Methods

#### 2.2.1 Synthesis of the Cross-Linked Phosphorylated Functional Cellulose

Phosphorylated FC was synthesized according to the procedure described elaborately in our previous studies.^1,47^ Nonetheless, due to the phosphorous groups in its structure, phosphorylated FC is a very hydrophilic substance that instantly swells to form a hydrogel when it comes into contact with water. Before using the phosphorylated FC in the column, the material was cross-linked with ECH to eliminate the hydrophilicity of the material, as it becomes hydrogel when filled into the column. To do this, the method applied directly for cellulose was given as follows:

Phosphorylated FC (10 g) was slurried in water (75 mL) containing sodium chloride (0.15 g) and ECH (0.2 mL, 0.4 mL, or 0.8 mL) in a glass beaker. Then, potassium hydroxide (0.6 g) dissolved in water (4 mL) was added slowly for 15 min to this slurry, and the mixture was stirred at 25 °C for 16 h. Afterward, the mixture was filtered, and the residue obtained was dried at 70 °C overnight.^51^ The reaction inputs of the applied method according to the desired degree of crosslinking are summarized in Table S1.

#### 2.2.2 Characterization of the Cellulose-Based Polymers

SEM was used to explore the textural properties of polymer materials with a Quanta 250 SEM equipment. The SEM photomicrographs of the materials whose free surfaces were coated with thin gold layers were taken in the 3.0–5.0 kV accelerating voltage range.

To determine the change in phosphorylated FC bond structures with and without crosslinking, a Shimazdzu FTIR-8400S spectrophotometer equipment was used to acquire IR spectra of 4000–400 cm^–1^ with a resolution of 4 cm^–1^ and 24 scans per sample. The KBr pellet approach was used to obtain IR spectra by scanning solid pellets containing roughly 2 mg of cellulosic material and 148 mg of spectroscopically pure KBr.

The samples’ Brunauer–Emmett–Teller (BET) surface area and pore size were evaluated using the Micromeritics Gemini V analyzer and the N_2_ adsorption–desorption method at 77 K. Analysis chamber containing samples was vacuumed up to a pressure of 20 mTorr for the first 2 h at 70 °C and then at 90 °C for 12 h.

A thermogravimetric analyzer (Shimadzu, TGA-51) was used to determine detectable thermal stability and heat capacity variations. Thermograms were acquired by dynamic heating under a nitrogen environment from 20 to 1000 °C with a heating rate of 5 °C/min.

#### 2.2.3 Equilibrium Swelling Test

Approximately 50 mg of phosphorylated FC and its cross-linked forms were equilibrated in 12 mL of distilled water at 25 °C for 72 h to investigate the swelling property. The hydrated polymer was obtained by centrifuging three times at 8 °C and 7000 rpm for 15 min and completely withdrawing the remaining water until it reached a constant weight. The swelling rate (*S*_w_) was calculated using eq 1, which includes the weight of the hydrated polymer (*W*_s_) and the dry weight of the polymer (*W*_d_):
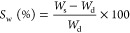
1

#### 2.2.4 Batch Studies for the Effect of Crosslinking on Lithium Sorption Efficiency

To investigate the effect of crosslinking on lithium sorption efficiency, the synthesized phosphorylated FC and its cross-linked forms with ECH at different ratios of 2-ECH (0.02 mL ECH/g FC), 4-ECH (0.04 mL ECH/g FC), and 8-ECH (0.08 mL ECH/g FC) were tested with a 12 g L^–1^ sorbent dose, a solution having 10 mg L^–1^ initial concentration of Li, via shaking at 30 °C for 24 h in a batch-mode operation. Li concentrations were determined with the ICP-OES equipment.

#### 2.2.5 Lithium Sorption–Desorption Studies in the Packed Bed Dynamic Column

**Figure 1:**
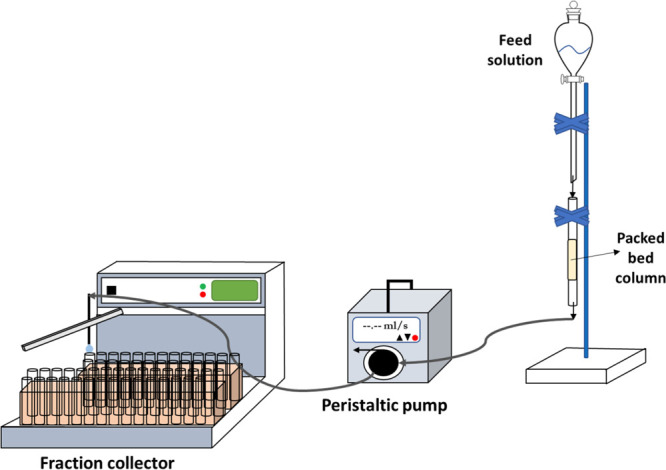
Schematic experimental setup representation of the column study.

A 0.7 cm diameter and 12 cm high glass column filled with cross-linked phosphorylated FC at a ratio of 0.04 mL ECH/g FC was used for the chromatographic separation of lithium from the aqueous solution. The schematic drawing of the experimental setup is given in Figure 1. First, the effect of the flow rate on lithium sorption was investigated by feeding the solution with an initial lithium concentration of 10 mg L^–1^ at different flow rates (0.25, 0.5, and 1.0 mL min^–1^) in a 1.5 cm bed height column from the top to bottom. In addition, the effect of bed height on lithium sorption was investigated with a flow rate of 0.5 mL min^–1^ at different bed heights (1.0, 1.5, and 2.0 cm). Samples (3 mL) were collected by time in lithium sorption experiments. Lithium desorption experiments were performed with a 5% by volume (0.51 M) H_2_SO_4_ solution at a flow rate of 0.12 mL min^–1^ by collecting a 2 mL fraction with the setup consisting of a peristaltic pump (SHENCHEN) and a fraction collector (BÜCHI C-660). The lithium concentration of the samples was determined with the help of a flame photometer (JENWAY PFP7) equipment.

Breakthrough curves showing the performance of fixed-bed column sorption were plotted as bed volume (BV) versus normalized concentration (*C*/*C*_0_), defined as the ratio of the effluent lithium concentration (*C*, mg/L) to the inlet lithium concentration (*C*_0_, mg L^–1^). BV (mL solution/mL sorbent) was calculated by eq 2:
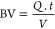
2where *t* is the operating time (min), *Q* is the flow rate of feed solution (mL/min), and *V* is the wet volume of sorbent packed in the column (mL).

## 3 Modeling of Packed Bed Column Dynamic Behavior

Modeling of experimental data is used to successfully design a column adsorption process and provide mathematical and quantitative approaches. Breakthrough curves for the adsorption of both inorganic ions and organic compounds in a fixed bed column are simulated with frequently used models such as the Thomas model, the Yoon–Nelson model, and the MDR model to describe the dynamic behavior.^49^ In this study, these three models were also evaluated to predict the rate and capacity parameters from breakthrough curves of Li sorption onto cross-linked phosphorylated FC.

### 3.1 Thomas Model

The Thomas model assumes the second-order reversible reaction kinetics, and the Langmuir isotherm is one of the most commonly used models to describe breakthrough curves. Specifically, it is convenient to predict the adsorption process in which internal and external diffusion are not rate-limiting steps.^52,53^ The Thomas model can be expressed as in eq 3:
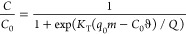
3where *K*_T_ [mL/(min mg)] is the Thomas rate constant and ϑ (mL) is the total volume of solution passing through the column at any given time. *q*_0_ is the sorption capacity (mg g^–1^) and *m* is the dry weight of the sorbent filled in the column. Other parameters have been defined previously.

The linearized form of the Thomas model can be written as in eq 4. *K*_T_ and adsorption capacity *q*_0_ values can be found from the slope and intercept of the graph between ln[(*C*_0_/*C*) – 1] and time (ϑ/*Q*), respectively:
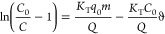
4

### 3.2 Yoon–Nelson Model

A relatively simple model focusing on the adsorption of gases or vapors on activated carbon was developed by Yoon–Nelson. The basic assumption made for this model to be applicable is the rate of decrease in the adsorption probability for each adsorbed molecule is proportional to the sorption probability of the adsorbed molecule and the probability of the molecule being adsorbed on the adsorbent.^54,55^ The Yoon–Nelson equation is given as follows:
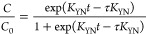
5where *K*_YN_ is the rate constant (min^–1^) and τ (min) is the time required for 50% of the molecule to be adsorbed to pass when the concentration (*C*, mg L^–1^) is half of the initial concentration (*C*_0_, mg L^–1^). *K*_YN_ and τ can be found from the slope and intercept of the graph [ln(*C*/(*C*_0_ – *C*)] versus *t*) plotted for the linearized form of the Yoon–Nelson model given in eq 6:
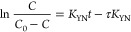
6

According to the Yoon–Nelson model, the adsorption capacity (*q*_0_, mg/g) can be calculated using eq 7. The model claims that the amount of lithium adsorbed by the sorbent is half of the initial lithium concentration passing through the packed column in the 2τ period.^56^
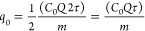
7

### 3.3 Modified Dose–Response Model

The MDR model is another simplified mathematical model used to evaluate the dynamic behavior of packed bed column adsorption data. This model reduces the error caused by using the Thomas model, especially at lower or higher periods of the breakthrough curve.^57^ The mathematical model is expressed in eq 8:
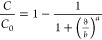
8

*a* and *b* are model constants, *b* specifies the output volume producing a half-maximum response, and *a* determines the slope of the regression function. From the *b* value, the value of the maximum solid-phase concentration of the solute (*q*_0_) can be calculated using eq 9:
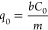
9

The model parameters were determined by fitting the experimentally obtained data with the help of MATLAB software using the nonlinear regression technique for the MDR model given in eq 8. The sum of the squares of the differences between the experimental data and the theoretical data (calculated from the models) was taken into account in error analysis to find the best-fitted model. The sum of squares of error (SSE) can be obtained from the following equation:
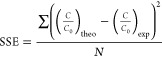
10(*C*/*C*_0_)_theo_ is the ratio of the outlet and inlet lithium concentration obtained from the model, (*C*/*C*_0_)_exp_ is the ratio of lithium concentration in the outlet and inlet water obtained from the experiment, and *N* is the total number of experimental points.

## 4 Results and Discussion

### 4.1 Characterization of Synthesized Cross-Linked Phosphorylated Functional Cellulose

#### 4.1.1 SEM Analysis

**Figure 2:**
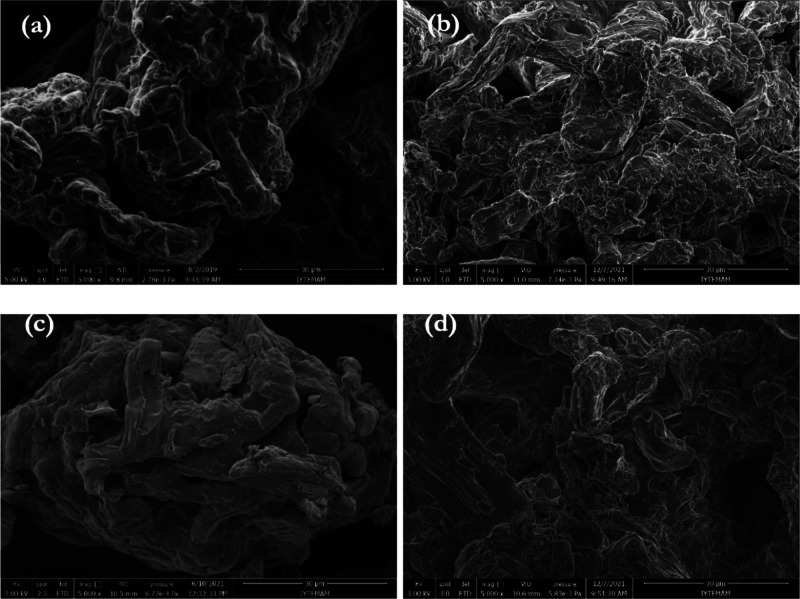
SEM surface morphology (magnification 5000x): (a) only phosphorylated, crosslinked with the ratio of (b) 0.02 mL ECH/g FC, (c) 0.04 mL ECH/g FC, and (d) 0.08 mL ECH/g FC phosphorylated functional cellulose.

Figure 2 shows the surface morphology of phosphorylated FC materials, where Figure 2a belongs to only phosphorylated FC fibers (without cross-linking). In contrast, Figure 2b–d belongs to cross-linked phosphorylated FC with a different cross-linking agent, ECH, with ratios of 0.02 mL, 0.04 mL, and 0.08 mL ECH/g, respectively. As the ratio of ECH increased, more porous structures were formed, although the network tended to agglomerate based on the formation of more crosslinks. SEM images support the formation of interconnected pores and capillary channels because it is known that at a higher ECH ratio, higher branching polymer chains are produced and an additional network is formed.^58^ In addition, ECH crosslinking in cellulose results in a macroporous interior, as evidenced by the BET analysis, the findings of which will be discussed elaborately later. The high ECH ratio increased the pore size, which can be explained by the slow cross-linking process at low temperatures with strong self-assembly of the free hydroxyl groups between cellulose chains.

#### 4.1.2 FTIR Analysis

**Figure 3:**
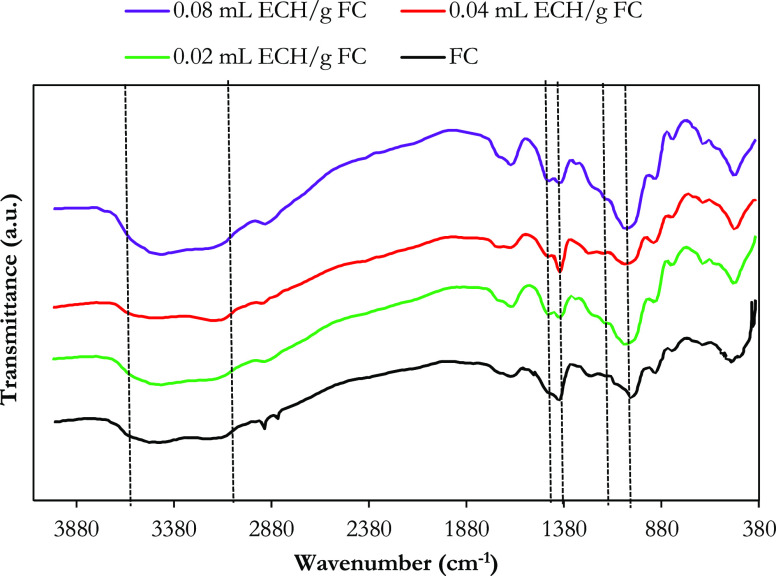
Micro-FTIR spectra of phosphorylated functional cellulose (FC) alone and its cross-linked forms with epichlorohydrin (ECH) at different rates (0.02 mL, 0.04 mL, and 0.08 mL/g FC).

The Fourier transform infrared (FTIR) spectroscopy is one of the characterization techniques used to verify cross-linked structures as it gives spectral properties from the formation of new binding arrangements or functional groups. Figure 3 illustrates FTIR spectra of phosphorylated FC alone and phosphorylated FC cross-linked with ECH at different ratios (0.02 mL, 0.04 mL, and 0.08 mL/g FC). The spectrum shows broadband attributable to intermolecular bonds such as hydroxyl (−OH) groups at 3000–3600 cm^–1^, C–H stretching at 2800–3000 cm^–1^, and O–H and C–H bending and C–O–H and C–O–C asymmetric stretching at 1400–1300 and 1000–1200 cm^–1^, respectively. Besides the relative signal intensities that grow and sharpen with higher ECH content, these bands are nearly identical to the various phosphorylated cellulose materials. Meanwhile, due to the stoichiometric excess of the crosslinker, self-crosslinking of the −OH group of ECH at the C2 position and hydrolysis of ECH may cause the weakening of these bands in places. In general, ECH is more likely to self-crosslink when the present −OH groups of the cellulose react or become sterically unavailable due to crosslinking and/or the fibrous character of the cellulose.^59^ This stepwise crosslinking process explains the lower density of the −OH band (3000–3600 cm^–1^) for this polymer compared to FC cross-linked with 0.08 mL ECH/g compared to other peak densities. Also, the C–O band at 1050–1200 cm^–1^, where new peaks appear due to the formation of new C–O bonds, is a marker for confirmation of successful crosslinking.

#### 4.1.3 BET Analysis

BET surface areas and Barrett–Joyner–Halenda (BJH) cumulative pore volume percentages for phosphorylated FC and its cross-linked forms at different ratios are listed in Table 1. In general, BET surface area values of less than 1 m^2^ g^–1^ were observed to decrease with the increasing crosslinking ratio for all modified materials. Because functional groups are attached to the surface of the material, FC and its cross-linked form in different proportions commonly have macropores. While the macropore volume in phosphorylated FC was 49.01% when the crosslinking ratio was increased from 2-ECH to 8-ECH, the percentage of macropores in the structure of the material increased from 58.80 to 73.25%. Relatively decreasing surface area and increasing macropores can be explained by the fact that the two free hydroxyl groups of the cellulose molecule bond with each other, as well as the hydrogen bonding effects of the phosphorous functional groups in the structure of the material with each other, and thus, that may also cause to decrease in the active sites where lithium can be retained.

**Table 1:** Pore Structure Parameters and BET Surface Areas for Phosphorylated Functional Cellulose (FC) Alone and Its Cross-Linked Forms with Epichlorohydrin (ECH) at Different Rates (0.02 mL, 0.04 mL, and 0.08 mL/g FC)

material	pore volume (%)			BET surface area (m^2^/g)	total pore volume (cm^3^/g)
	micropore (<2 nm)	mesopore (2–50 nm)	macropore (>50 nm)		
FC	17.75	33.24	49.01	0.95	0.0004
2-ECH	22.89	18.31	58.80	0.79	0.0003
4-ECH	6.23	25.09	68.68	0.76	0.0006
8-ECH	7.44	19.31	73.25	0.73	0.0005

#### 4.1.4 TGA Analysis

**Figure 4:**
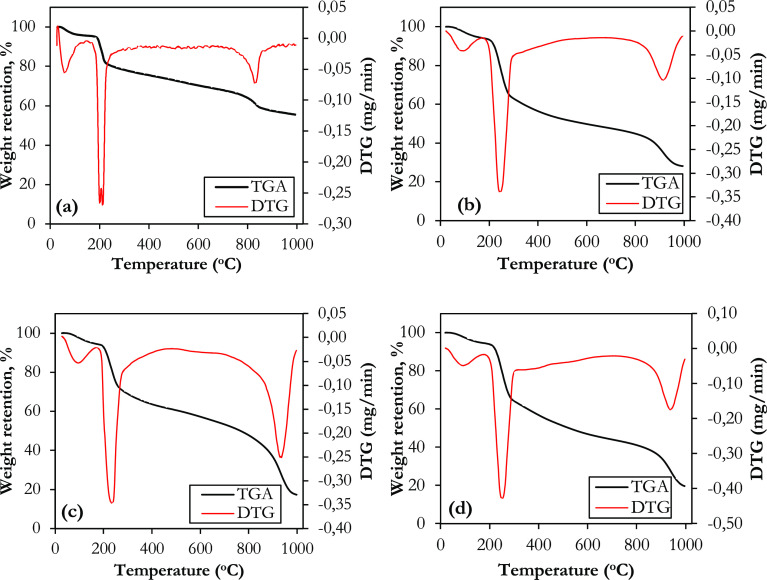
Thermogravimetric analysis curves of functional cellulose (a) phosphorylated only, cross-linked at (b) 0.02 mL ECH/g FC, (c) 0.04 mL ECH/g FC, and (d) 0.08 mL ECH/g FC ratios.

TGA is one of the sensitive characterization methods for observing crosslinking reactions due to measurable differences in thermal stability and heat capacity of pure and cross-linked materials.^60^ The TGA results of phosphorylated FC that is not cross-linked and cross-linked with ECH at different ratios and the thermal stability profile of thermal degradation events between 20 and 1000 °C are shown in Figure 4. Basically, one minor and two major weight losses are observed for all materials. Data on these findings are given in Table 2. Minor weight losses of approximately 2–3% observed in raw and cross-linked phosphorylated FC materials in the temperature range of 86–95 °C are due to the evaporation of water molecules adsorbed by the materials. Depending on the primary major weight losses, thermal degradation events were recorded between 200 and 250 °C. Non-crosslinked phosphorylated cellulose decomposed at ∼200 to 213 °C, while the cross-linked polymers showed offset values: 2-ECH (∼245 °C), 4-ECH (∼236 °C), and 8-ECH (∼250 °C). These results indicate that the main degradation events for cross-linked polymers occur at higher temperatures than for no cross-linked phosphorylated cellulose. The situation at temperatures where primary major weight loss is observed indicates that the glycosidic bonds in the cellulose structure are degraded. On the other hand, phosphorus-containing functional groups are known to increase the thermal degradation reaction of the cellulosic polymer (and thus decrease the heat resistance of the material) but instead significantly increase the carbonization (and, therefore, the flame resistance).^61^ Furthermore, cellulosic polymers containing phosphoryl compounds exhibit flame retardant qualities, according to the literature.^62−64^ Based on this, the secondary major weight losses observed in all materials in the temperature range of 830–940 °C are the temperatures at which phosphorous functional groups decompose. As the crosslinking ratio increased, the weight losses of the materials increased, but the maximum decomposition temperature shifted from 832 to 937 °C. 8-ECH exhibited higher thermal durability due to the higher crosslinking ratio. The lower temperatures where primary major weight losses were observed are attributed to the covalent bond interaction of the polymer framework. In contrast, the higher temperature event secondary to major weight losses can be explained by the reduction in heat capacity and hydrogen bond interactions between the cellulose chains. This trend also coincides with structural effects from crosslinking, such as the macropore formation and related changes in heat capacity observed for similar types of cross-linked materials.^59^

**Table 2:** TGA Findings of Crude and Cross-Linked Phosphorylated Functional Cellulose

material	minor weight loss		primary major weight loss		secondary major weight loss		the mass remained at the end of 1000 °C (%)
	temperature (°C)	weight loss (%)	temperature (°C)	weight loss (%)	temperature (°C)	weight loss (%)	
FC	91	3.32	200–213	11.37	832	24.52	55.40
2-ECH	86	2.10	245	18.76	912	43.76	28.04
4-ECH	95	2.20	236	17.50	933	52.20	17.30
8-ECH	91	2.15	250	17.97	937	52.08	19.52

#### 4.1.5 Equilibrium Swelling Properties of Hydrophilic and Cellulosic Materials

Lists of the findings of equilibrium swelling tests of phosphorylated FC and its cross-linked forms are listed in Table S2. The results show that phosphorylated FC has a higher swelling ratio than cross-linked polymers, which is consistent with independent expectations. As the ECH increased, the material turned into a more rigid structure and the swelling ratio decreased. This decreasing trend can be explained by less passage of water into the fiber domains of cellulose. It has been observed that the change in the swelling ratio is more minor when the cross-linker ratio is increased from 4-ECH to 8-ECH, compared to the variation in the swelling ratio from 0 to 2-ECH and from 2-ECH to 4-ECH. Based on this, the optimum crosslinking rate was determined as 4-ECH (0.04 mL ECH/g FC). Then, the effect of crosslinking on lithium sorption was also investigated to strengthen the selection of the optimum crosslinking ratio.

### 4.2 Effect of Cross-Linking on Lithium Sorption Efficiency

**Figure 5:**
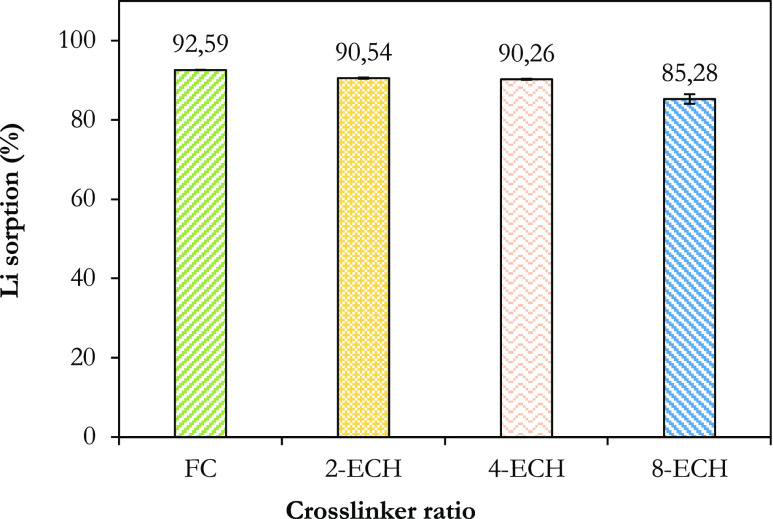
Comparison of lithium sorption efficiencies of phosphorylated functional celluloses without crosslinking and with crosslinking via epichlorohydrin at different ratios.

Removing contaminants from wastewater at a large scale economically would open up new technological frontiers, and natural carbohydrate polymeric materials and tunable and sensitive materials are in great demand.^65,66^ In Figure 5, the lithium sorption percentages of phosphorylated FC without crosslinking and crosslinking at different ratios were compared via a batch-mode operation. According to the findings, the lithium sorption of phosphorylated FC without crosslinking was 92.59%, and the yield decreased gradually as the cross-linker ratio increased. Lithium sorption efficiency was 85.25% at the highest cross-linker ratio (0.08 mL ECH/g FC). This situation can be explained by the fact that the two free hydroxyl groups of cellulose are bonded to each other, as well as the decrease in the active sites where lithium can be retained due to the hydrogen bonding effects of the phosphorous functional groups in the structure of the material with each other. As reported in our previous study,^47^ according to the crosslinking structure of phosphorylated cellulose and its combined stoichiometry, a P/Li atom ratio was obtained at approximately 1.03 from XPS measurements, indicating the presence of these phosphorous moieties on the adsorbent surfaces because one Li^+^ is exchanged with H^+^ in the functional group and the expected ion exchange mechanism is given in eq 11:
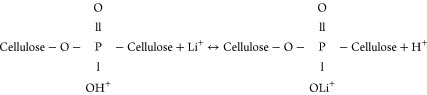
11

Hence, considering both the swelling ratio and lithium sorption efficiency of the material, the optimum cross-linker ratio was determined as 0.04 mL ECH/g FC and phosphorylated FC cross-linked at this ratio was used in further column studies.

### 4.3 Packed Bed Column Studies of Cross-Linked Phosphorylated Functional Cellulose

The breakthrough time that gives the operating life of the adsorbent in a single adsorption process and breakthrough capacity are significant parameters that entirely affect the performance of the column adsorption process. The breakthrough time is directly related to the ratio of the outlet concentration to the inlet concentration. Namely, the value of 0.05 as the breakthrough time is taken as the ratio between the effluent and the feed concentrations for the removal of heavy metal ions from water in fixed bed operations. However, because it was aimed to recover a valuable metal in this study, based on the literature, the breakthrough time (*t*_b_, min) was defined as the time required for the lithium extraction rate to decrease to 0.6 and is expressed by eq 12.^67^ In other words, the time when the solution with an initial concentration of 10 mg L^–1^ is obtained as approximately 4 mg/L at the effluent was accepted as the breakthrough time. The breakthrough capacity and column utilization degree were calculated according to this assumption:
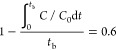
12

#### 4.3.1 Effect of Flow Rate on Lithium Sorption Capacity in a Packed Bed Column

**Figure 6:**
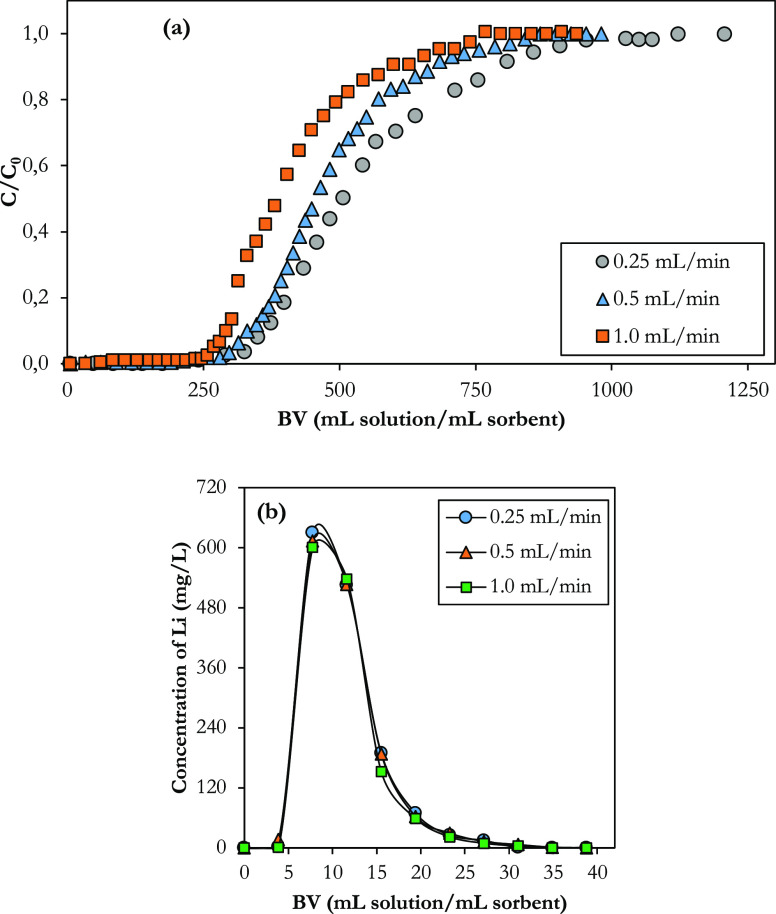
(a) Breakthrough curves and (b) elution curves of the column filled with cross-linked phosphorylated functional cellulose at 0.04 mL ECH/g FC at different flow rates (*C*_0_ = 10 mg L^–1^, *T* = 25 °C, bed height = 1.5 cm, ϑ = 0.25–1.0 mL min^–1^).

Flow rate is an important parameter to determine the effectiveness of sorbents in the continuous treatment of wastewater. The breakthrough curves of lithium sorption obtained with varying flow rates at the constant inlet lithium concentration (10 mg L^–1^) and bed height (1.5 cm; 0.58 mL sorbent) are given in Figure 6a. The trend of the breakthrough curves has been obtained as commonly observed in liquid phase adsorption, where intraparticle diffusion is the rate-limiting transport process.^68−70^ The effect of the feed flow rate on the breakthrough point was particularly pronounced when it decreased from 1.0 to 0.25 mL min^–1^, with an increase in the flow rate found to increase the sharpness of the breakthrough curve. Also, increasing the flow rate shifted the breakthrough curves significantly from right to left, indicating that the service time of the fixed bed is reduced. The higher the flow rate, the earlier the release. Table 3 compares and summarizes the study results such as capacity, elution efficiency, and column utilization degree obtained using cross-linked phosphorylated FC at different flow rates. As the flow rate increases from 0.25 to 1.0 mL min^–1^, the BV required for release has fallen from 477 mL solution/mL sorbent to 386 mL solution/mL sorbent, as there is a decrease in mass transfer rate and faster lithium passes through the column. The breakthrough capacities at the three flow rates (0.25, 0.5, and 1.0 mL min^–1^) were 25.82, 23.60, and 18.81 mg g^–1^, respectively, while the total capacities were 33.56, 30.15, and 25.54 mg g^–1^. The decrease in capacity as the flow rate increases can be explained by the insufficient residence time of lithium ions in the column and the intraparticle diffusion to the reaction sites, which limited the mass transfer rate at a high flow rate.^71,72^ Operating at a flow rate of 0.25 mL min^–1^, the BV for the total capacity was 1260, that is, 730 mL of a solution containing 10 mg L^–1^ Li took approximately 2900 min (48 h) to saturate the material. Compared to the previously published capacity of phosphorylated FC, the material exhibited a much higher lithium uptake capacity when used in the column, despite its cross-linking structure. As a result of batch sorption studies, the maximum lithium retention capacity was determined as 9.60 mg/g.^47^ In addition, lithium elution curves for all sorption tests carried out at different flow rates are plotted in Figure 6b. The desorption rate was fast and slowed down with a decrease in the effluent Li concentration from higher than 625 mg L^–1^ to lower than 1 mg L^–1^ and, subsequently, zero in a few fractions and time, resulting in *ca*.100% elution efficiencies at all studied flowrates. Only 15.75 mL of 5% H_2_SO_4_ solution (0.51 M) was required to desorb approximately 100% of Li from the saturated sorbent to do the desorption. This shows that the lithium retained by the material can be easily and completely recovered. Li in the concentrated acid can be purified and precipitated for further use.

**Table 3:** Comparison of Fixed Bed Performance at Different Flow Rates

	0.25 mL min^–1^	0.5 mL min^–1^	1.0 mL min^–1^
breakthrough capacity, mg Li/mL sorbent	4.8	4.4	3.5
breakthrough capacity, mg Li/g-sorbent	25.8	23.6	18.8
BV at breakthrough point, mL solution/mL sorbent	477	459	386
total capacity, mg Li/mL sorbent	6.3	5.7	4.8
total capacity, mg Li/g-sorbent	33.6	30.2	25.5
BV at total capacity, mL solution/mL sorbent	1260	935	857
degree of column utilization, %	76.9	78.3	73.7
elution efficiency, %	∼100	∼100	∼100

#### 4.3.2 Effect of Bed Height on Lithium Sorption Capacity in a Packed Bed Column

**Figure 7:**
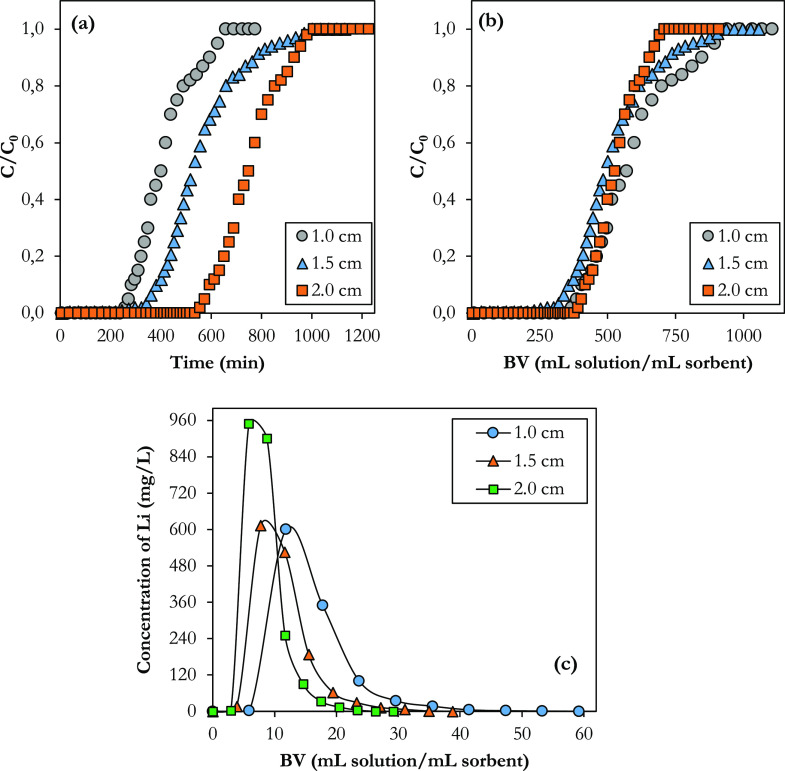
(a) Breakthrough curves; *C*/*C*_0_ vs time, (b) breakthrough curves; *C*/*C*_0_ vs bed volumes, (c) elution curves of the column filled with cross-linked phosphorylated functional cellulose at 0.04 mL ECH/g FC at different bed heights (*C*_0_ = 10 mg L^–1^, *T* = 25 °C, ϑ = 0.5 mL min^–1^, bed height = 1.0–2.0 cm).

At a constant flow rate of 0.5 mL min^–1^ and a constant feed concentration of 10 mg L^–1^, the effect of bed height on breakthrough curves, *C*/*C*_0_ versus time, is demonstrated in Figure 7a. Phosphorylated FC was filled in the column at three different heights, 1.0, 1.5, and 2.0 cm, equivalent to wet sorbent volumes of 0.38, 0.58, and 0.77 cm^3^, respectively. A higher bed height altered the breakthrough curve to the right, lengthening the period between breakthrough and saturation. This result could be explained by the increased sorbent mass providing more accessible active sites for sorption at the higher bed height. As the bed height increased from 1.0 to 2.0 cm, the solution that passed through the column at the breakthrough time increased from 196 to 385 mL. As shown in Figure 7b, the three curves closely overlapped when the breakthrough curves were replotted using the number of BVs to abscissa, revealing that the increase in bed height or sorbent volume had almost no effect on the dynamic sorption of Li. As given in Table 4, the similar sorption capacity at the breakthrough and total saturation points and the similar degree of column utilization further supported this evidence. The breakthrough times at the three different bed heights (1.0, 1.5, and 2.0 cm) were 362, 491, and 710 min, respectively, and the total capacities were 30.9, 30.2, and 31.7 mg g^–1^. Moreover, the degree of column utilization for each bed height was 83.8, 78.3, and 81.1%, respectively. Recently, the effect of bed height on lead removal efficiency was investigated and no impact was observed on sorption capacity with different bed heights.^49^ Overall, these findings proved that increasing the bed height enhanced the treated water volume for lithium recovery without affecting the sorption capacity, paving the way for the widespread use of phosphorylated FC in dynamic column systems. On the other hand, elution curves of the column filled with cross-linked phosphorylated cellulose obtained by plotting the concentration of Li in effluent 5% H_2_SO_4_ against the BVs at the studied bed heights are illustrated in Figure 7c. The concentrated lithium was the highest for 2.0 cm bed height, depending on the highest total sorption capacity. Because the sorbent is easily regenerated with the acid, almost 100% elution efficiencies were also obtained for each bed height.

**Table 4:** Comparison of Fixed Bed Performance at Different Bed Heights

	1.0 cm	1.5 cm	2.0 cm
breakthrough capacity, mg Li/mL sorbent	4.8	4.4	4.8
breakthrough capacity, mg Li/g-sorbent	25.9	23.7	25.7
breakthrough time, min	362	491	710
total capacity, mg Li/mL sorbent	5.8	5.7	5.9
total capacity, mg Li/g-sorbent	30.9	30.2	31.7
total saturation time, min	659	1001	1007
degree of column utilization, %	83.8	78.3	81.1
elution efficiency, %	∼100	∼100	∼100

### 4.4 Modeling of Breakthrough Curves

The breakthrough curves of Li at different flow rates and bed heights were investigated with three commonly used models, Thomas, Yoon–Nelson, and MDR, whose parameters and correlation coefficients (*R*^2^) and SSE values are tabulated in Tables 5 and S3, respectively, and the figures representing theoretical and experimental data plots for each model at different flow rate and bed height are given in Figure S1. According to lower SSE values (0.0002–0.0007) and higher correlation coefficient, *R*^2^ (0.996–0.999), reproducing better fitting of experimental data with the theoretical ones, the MDR model has better described the dynamic behavior of Li sorption using phosphorylated FC in a packed bed column.

**Table 5:** Breakthrough Model Parameters of Li Sorption on Phosphorylated Functional Cellulose

	parameters		model							
exp. no.	flow rate (mL/min)	bed height (cm)	Thomas		Yoon–Nelson			MDR		
			*K*_T_	*q*_0_	*K*_YN_	τ	*q*_0_	*a*	*b*	*q*_0_
1	0.25	1.5	0.458	32.5	0.008	998.1	29.3	5.54	0.30	29.88
2	0.5	1.5	1.782	25.0	0.015	495.9	25.4	6.52	0.27	25.31
3	1.0	1.5	2.018	20.1	0.021	206.0	20.1	6.05	0.23	20.33
4	0.5	1.0	1.880	31.0	0.018	398.7	30.4	6.59	0.21	30.25
5	0.5	2.0	1.330	28.4	0.013	753.3	28.4	10.7	0.40	28.05

#### 4.4.1 Thomas Model Analysis

Thomas model analysis reveals that the high mass transfer rate led to the values of *K*_T_ increasing from 0.458 to 2.018 mL min^–1^ mg^–1^ as the flow rate increased from 0.25 to 1.0 mL min^–1^. While increasing the flow rate reduced the value of *q*_0_ up to 20.1 mg g^–1^, this was primarily due to the insufficient time for Li diffusion and adsorption. In terms of bed depth, the results showed that the value of *K*_T_ decreased from 1.880 to 1.330 mL min^–1^ mg^–1^ as bed depth increased from 1.0 to 2.0 cm, which can be attributed to the longer contact time at greater bed depths. The value of *q*_0_, on the other hand, was observed to increase as the bed depth was increased because the adsorption sites increased. This suggested that the adsorption rate would be faster at a higher flow rate and lower bed height. Besides, these findings are consistent with those found in the literature.^73,74^ Nonetheless, the model cannot precisely trace the sorption of Li in the column or estimate the minimum height of the adsorption front, allowing for a better understanding of performance. The application of the MDR approach can overcome such a flaw.

#### 4.4.2 Yoon–Nelson Model Analysis

Previous studies have effectively employed the Yoon–Nelson model to describe pollutant adsorption in a fixed-bed design.^75^ When the Yoon and Nelson model was applied to the experimental data, *K*_YN_ and τ values were obtained that accurately characterized the packed bed sorption of Li onto the phosphorylated FC. Decreasing the flow rate from 1.0 to 0.25 mL min^–1^ with the same column height resulted in an increase of τ as a longer time is clearly required to saturate the material with Li due to providing sufficient contact time at lower flow rates. However, for 1.0 mL min^–1^ τ fell to 206 min, owing to the fact that a higher flow worsens the mass transfer process (e.g., kinetics and capacity), reducing the time required to recover 50% of Li. When the column height was increased from 1.0 to 2.0 cm, the time to obtain 50% of retention for Li increased from 398.7 to 753.3 min, which is appropriate because the accessible sorbent sites increased correspondingly with column height, which is also supported by the results of previous studies.^76,77^

#### 4.4.3 Modified Dose–Response (MDR) Model Analysis

The MDR model shows that as the flow rate increased from 0.25 to 1.0 mL min^–1^, the sorption capacity per unit mass of phosphorylated FC (*q*_0_) decreased from 29.88 to 20.33 mg g^–1^. When the area of the breakthrough curve of Li for 1.0 and 2.0 cm bed heights at 0.5 mL min^–1^ is similar, the column capacity appears to have a trend opposite to values of *q*_0_ in the MDR model because the column capacity considers the area of the breakthrough curve that is dominated by the saturation time. In addition, the values of b decreased with the increase in the flow rate and increased with an increase in the bed height.

To sum up, the SSE produced from the MDR model was much lower than that obtained from the Yoon–Nelson and Thomas models. As a result, the MDR model outperforms the Yoon–Nelson and Thomas models in predicting the adsorption behavior of phosphorylated FC for lithium sorption from water by chromatographic separation.

## 5 Conclusions

Natural carbohydrate polymeric materials and tunable and sensitive materials are in great demand for removing contaminants and recovering valuable minerals economically at large-scale operations such as dynamic column processes. Phosphorylated FC-ECH polymers were synthesized with variable crosslinking ratios (0.02, 0.04, and 0.08 mL ECH/g FC) and characterized via SEM, FTIR, BET, and TGA. Phosphorylated FC was modified via crosslinking with ECH to obtain more water stable and thermally more robust form and employed in a packed bed column to examine its continuous Li recovery capability. The feed flow rate influenced the dynamic recovery of Li by phosphorylated FC, and a lower flow rate was preferred for greater sorption capacity and a better degree of column utilization. As the flow rate increases from 0.25 to 1.0 mL min^–1^, the BV required for release has fallen from 477 mL solution/mL sorbent to 386 mL solution/mL sorbent, as there is a decrease in mass transfer rate and faster lithium passes through the column. The breakthrough capacities at the three flow rates (0.25, 0.5, and 1.0 mL min^–1^) were 25.82, 23.60, and 18.81 mg g^–1^, respectively, while the total capacities were 33.56, 30.15, and 25.54 mg g^–1^. Meanwhile, Park et al.^78^ evaluated the applicability of HMO as a sorbent for lithium recovery from seawater having 0.17 mg/L of Li in a fixed bed column and found 3.12 mg/g of total capacity with sorbent dose 5.0 g, superficial velocity 0.15 cm/s for 3 days of operation. The change in the bed height demonstrated almost no effect on sorption capacity. Increasing the bed height enhanced the treated water volume for lithium recovery without affecting sorption capacity. Although the breakthrough times at the three different bed heights (1.0, 1.5, and 2.0 cm) were 362, 491, and 710 min, respectively, and the total capacities were 30.85, 30.15, and 31.68 mg g^–1^. Thus, the widespread use of phosphorylated FC in dynamic column systems was clarified. The Thomas, Yoon–Nelson, and MDR models were used to examine the breakthrough curves, and the experimental data fitted in the order: MDR > Yoon–Nelson > Thomas. It was discovered that both the Thomas and Yoon–Nelson models might be utilized to forecast the lithium recovery process by comparing the correlation coefficients (*R*^2^) and SSE values under different operating conditions. However, the MDR model best estimates how effluent concentration changes over time during lithium sorption. As a result, due to its adaptability for a continuous system, phosphorylated FC strengthened with crosslinking via ECH was discovered to be a potential sorbent for the extraction of lithium from aqueous solutions in a dynamic packed bed column. Indeed, the cross-linked phosphorylated cellulose-based adsorbent is an improvement because the study’s findings suggested that prospective low-cost adsorbents might be made from various lignocellulosic biomass wastes with a high cellulose content and also paved the door for lithium recovery from natural water sources such as geothermal water by continuous processes because the Li sorption capacity in the presence of Na^+^, K^+^, Ca^2+^, and Mg^2+^ ions were investigated additionally in our previous batch mode of operation studies.^1,47^ The results showed that the functional group attached to the material exhibited more affinity towards Li among other monovalent cations. On the other hand, in the presence of Ca^2+^ and Mg^2+^, they highly competed with monovalent ions since they have a higher valence and atomic radius as expected, but the material still showed a considerable lithium sorption capacity.

## Supporting Information Available

ECH/FC ratio for crosslinking reaction; equilibrium swelling data of FC and its cross-linked forms; correlation coefficients and SSE values of breakthrough models; and Graphs for model fittings to experimental data (PDF)
