# Supplementary material for: Cross-Linked Phosphorylated Cellulose as a Potential Sorbent for Lithium Extraction from Water: Dynamic Column Studies and Modeling
Source: ACS Omega. 2022 Oct 21;7(43):38957–68. doi: 10.1021/acsomega.2c04712 (PMC9631899; doi:10.1021/acsomega.2c04712)
Supplement: Supplementary file 1 — ao2c04712_si_001.pdf [file ao2c04712_si_001.pdf]

Supporting Information

Cross-linked Phosphorylated Cellulose as Potential Sorbent for Lithium Extraction from Water: Dynamic Column Studies and Modeling

Yaşar Kemal Recepoğlu[1] and Aslı Yüksel[1,2*]

[1]Izmir Institute of Technology, Department of Chemical Engineering, 35430, Urla, Izmir, TURKEY

[2]Izmir Institute of Technology, Geothermal Energy Research and Application Center, 35430, Urla, Izmir, TURKEY

**asliyuksel@iyte.edu.tr ; Tel: +90 232 750 6609*

Supplementary Tables and Figures

Table S1. Amount of phosphorylated functional cellulose and epichlorohydrin in the synthesis of cross-linked materials

Reaction inputs	2-ECH*	4-ECH**	8-ECH***
Amount of functional material, g	10	10	10
Epichlorohydrin (ECH), mL	0.2	0.4	0.8

*0.02 mL ECH/g functional material; **0.04 mL ECH/g functional material; ***0.08 mL ECH/g functional material

Table S2. Equilibrium swelling data of phosphorylated functional cellulose and its cross-linked forms in water at 25°C

Material	W_s (g)	W_d (g)	Swelling ratio (%)
FC	0.42	0.0506	727.1
2-ECH	0.37	0.0508	626.2
4-ECH	0.30	0.0506	498.2
8-ECH	0.27	0.0508	438.4

Table S3. Correlation coefficients and SSE values of breakthrough models

	Parameters		Thomas		Yoon-Nelson		MDR	
Exp. No.	Flow rate (mL/min)	Bed height (cm)	R^2	SSE	R^2	SSE	R^2	SSE
**1**	0.25	1.5	0.96	0.0033	0.98	0.0034	0.99	0.0003
**2**	0.5	1.5	0.99	0.0013	0.99	0.0009	0.99	0.0002
**3**	1.0	1.5	0.98	0.0094	0.99	0.0053	0.99	0.0007
**4**	0.5	1.0	0.99	0.0015	0.99	0.0012	0.99	0.0005
**5**	0.5	2.0	0.98	0.0004	0.99	0.0004	0.99	0.0002

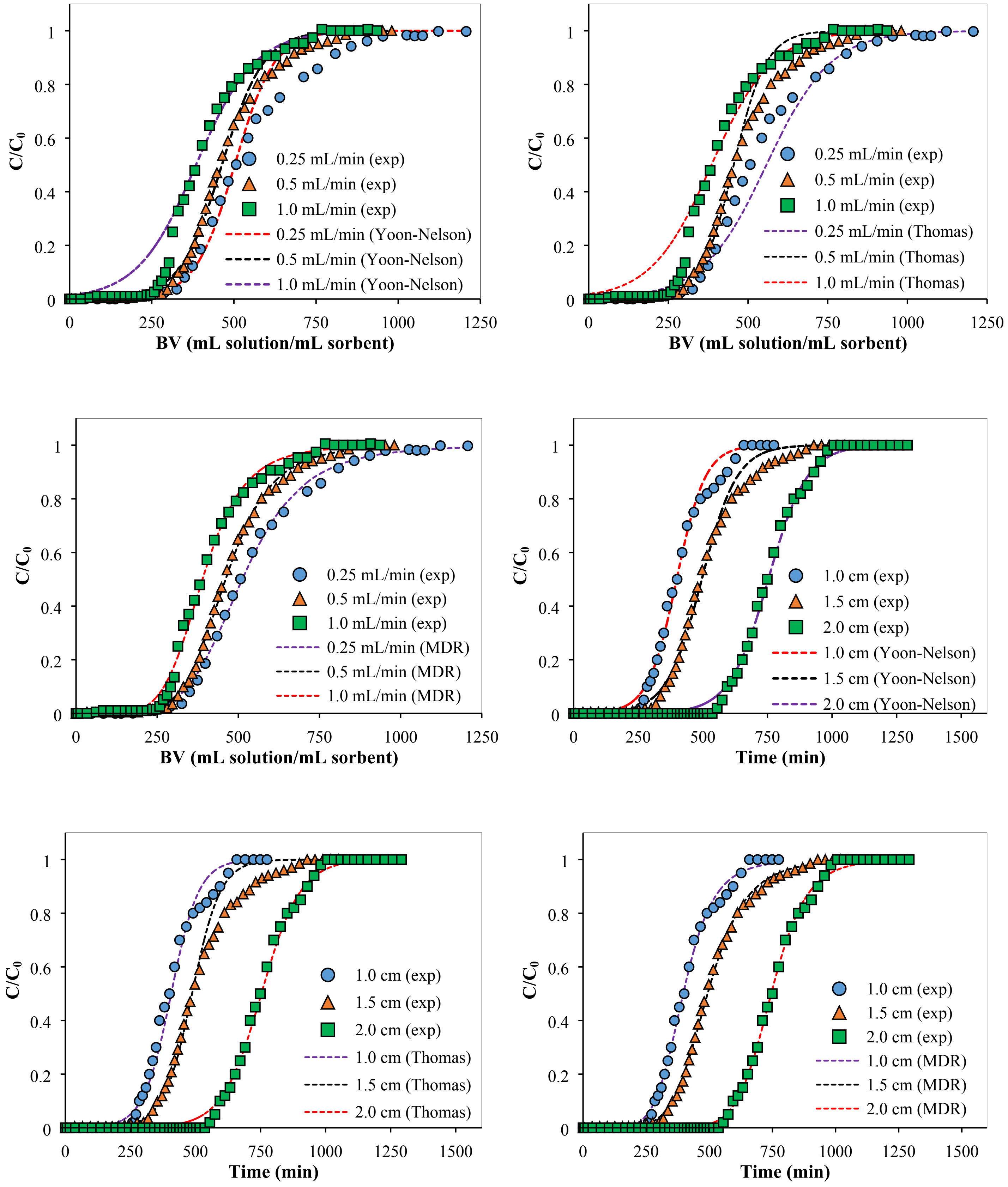

Figure S1. Model fittings of experimental data obtained from column study under various operating conditions
